# A Black Bean Story

**Published:** 1859-06

**Authors:** 


### A BLACK BEAN STORY.

On a mid October day, 1858, a little girl aged four years took a small shining hard black bean between her fingers, and put it to her nose to smell, it disappeared. She said it was in her nose; a surgeon was called who upon examination, declared that it could not be there. The mother was quieted, the child soothed, and ran about as if nothing had happened, maintaining her high and vigorous health. On the afternoon of the eight of May following, it was discharged from her nose, by the slight blow a child can make, into a handkerchief; the moment she saw it, she cried the bean ! the bean ! and ran with great glee to show it to her parents; it was now dingy, soft, shrivelled, and we found on measuring that it was two-eighths of an inch thick, four-eighths broad, and five eighths long. During these seven months the child never complained of any thing in the direction of the accident, except an occasional smarting of the eyes. On the previous evening, she had been soused head and ears under water in a bath tub, and the struggles incident to an operation of that kind, most likely dislodged it, for in the middle of the night she waked up with a startle and a cry, and after some throwing herself about, went to sleep in an hour, but was fretful all next day until the discharge of the bean, when her accustomed joyousness returned. Lesson—Don't give beans to children for playthings.

